# Effects of fetuin-A-containing calciprotein particles on posttranslational modifications of fetuin-A in HepG2 cells

**DOI:** 10.1038/s41598-021-86881-0

**Published:** 2021-04-05

**Authors:** Hideki Uedono, Katsuhito Mori, Akinobu Ochi, Shinya Nakatani, Yuya Miki, Akihiro Tsuda, Tomoaki Morioka, Yuki Nagata, Yasuo Imanishi, Tetsuo Shoji, Masaaki Inaba, Masanori Emoto

**Affiliations:** 1 Department of Metabolism, Endocrinology and Molecular Medicine, Osaka City University Graduate School of Medicine, Osaka, Japan; 2 Department of Nephrology, Osaka City University Graduate School of Medicine, Osaka, Japan; 3 Department of Vascular Medicine, Osaka City University Graduate School of Medicine, Osaka, Japan; 4 Vascular Science Center for Translational Research, Osaka City University Graduate School of Medicine, Osaka, Japan

**Keywords:** Renal replacement therapy, Chronic kidney disease, Phosphorus metabolism disorders, Endocrine system and metabolic diseases, Chronic inflammation

## Abstract

Fetuin-A is an inhibitor of ectopic calcification that is expressed mainly in hepatocytes and is secreted into the circulation after posttranslational processing, including glycosylation and phosphorylation. The molecular weight (MW) of fully modified fetuin-A (FM-fetuin-A) is approximately 60 kDa in an immunoblot, which is much higher than the estimated MW by amino acid sequence. Under conditions of calcification stress such as advanced stage chronic kidney disease, fetuin-A prevents calcification by forming colloidal complexes, which are referred to as calciprotein particles (CPP). Since the significance of CPP in this process is unclear, we investigated the effect of synthetic secondary CPP on the level of FM-fetuin-A in HepG2 cells. Secondary CPP increased the level of FM-fetuin-A in dose- and time-dependent manners, but did not affect expression of mRNA for fetuin-A. Treatment with O- and/or N-glycosidase caused a shift of the 60 kDa band of FM-fetuin-A to a lower MW. Preincubation with brefeldin A, an inhibitor of transport of newly synthesized proteins from the endoplasmic reticulum to the Golgi apparatus, completely blocked the secondary CPP-induced increase in FM-fetuin-A. Treatment with BAPTA-AM, an intracellular calcium chelating agent, also inhibited the CPP-induced increase in the FM-fetuin-A level. Secondary CPP accelerate posttranslational processing of fetuin-A in HepG2 cells.

## Introduction

Fetuin-A is a multifunctional protein that is mainly expressed in the liver^1–3^ and is secreted into the bloodstream after posttranslational modifications such as N-linked and O-linked glycosylations and serine phosphorylations. The molecular weight (MW) of fully-modified fetuin-A (FM-fetuin-A) is approximately 60 kDa on an immunoblot. One prominent function of FM-fetuin-A is its capacity to inhibit ectopic calcification^1–3^. Fetuin-A can protect against mineralization by forming colloidal complexes, which are referred to as calciprotein particles (CPP) and consist mainly of calcium, phosphate and fetuin-A^4,5^. Early calcification stress densifies calcium and phosphate ions to form primary CPP, which are stabilized by fetuin-A and contain amorphous calcium phosphae. Upon further calcification stress, primary CPP transform into larger secondary CPP with stable mineral cores covered by densely packed fetuin-A monolayers. The secondary CPP are spindle-shaped and 100–200 nm in diameter^5^.

The inhibitory role of fetuin-A against unfavorable calcification is clear, but the significance of the subsequently formed CPP is not fully understood. Synthetic secondary CPP induce inflammatory cytokines such as tumor necrosis factor (TNF)-α and interleukin (IL)-1β in a murine macrophage-like cell line^6^, and secondary CPP also promote calcification of vascular smooth muscle cells (VSMCs)^7^. In contrast, recent work has shown that the possible physiological role of small CPP may be equivalent to that of primary CPP or precursors using bisphosphonate^8^. These smaller CPP induce stronger fibroblast growth factor-23 expression in osteoblasts, compared to inorganic phosphate or larger CPP. Therefore, small CPP may be intrinsic carriers of dietary phosphate from intestine to bone^4^. These findings suggest that CPP might have physiological and pathological consequences.

The clinical relevance of fetuin-A is of interest in patients with advanced chronic kidney disease (CKD), which is often accompanied by vascular calcification. Previous studies have associated fetuin-A deficiency with a poor prognosis in patients undergoing dialysis^9,10^, and elevated levels of CPP have been observed in patients with CKD^11,12^. Given the inferred relationships among decreased fetuin-A, increased CPP, and accelerated vascular calcification in patients with advanced CKD, there may be a feedback loop between CPP and fetuin-A. Therefore, the objective of this study was to investigate the effects of secondary CPP on fetuin-A expression in the human hepatoma HepG2 cell line.

## Results

### Formation of CPP and effects of synthetic secondary CPP on the FM-fetuin-A level in HepG2 cells

**Figure 1:**
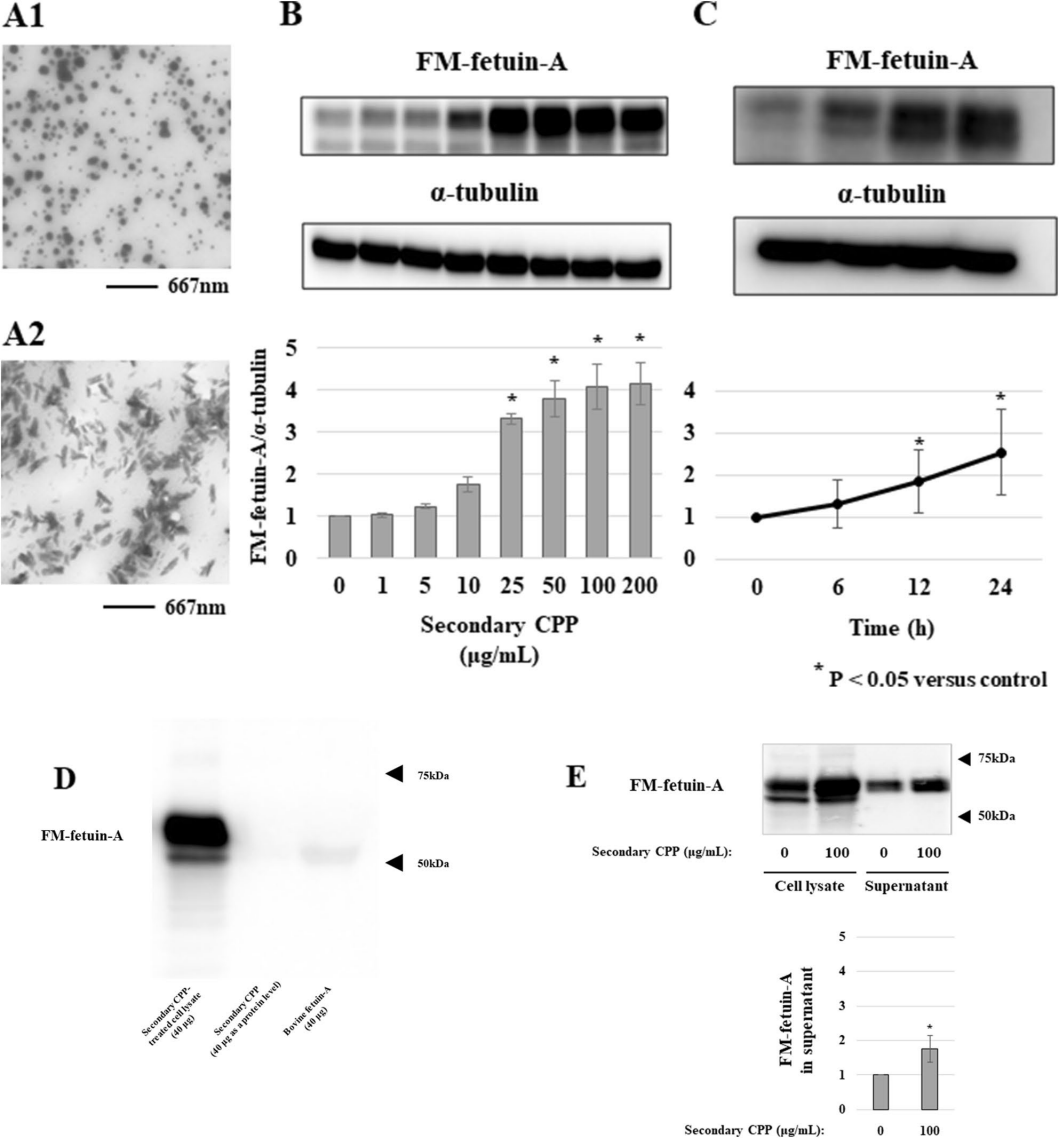
TEM imaging of synthetic CPP and effects of secondary CPP on FM-fetuin-A in HepG2 cells. (**A1**) After 1.5 h of incubation of CPP solution, small spherical-shaped CPP (primary CPP) were formed. (**A2**) At 24 h, larger crystalline-like CPP (secondary CPP) were observed. (**B**) HepG2 cells were treated with different concentrations (0, 1, 5, 10, 25, 50, 100, and 200 μg/mL) of synthetic secondary CPP for 24 h. Immunoblots of cell lysates were probed with fetuin-A (**B**, upper panel) and α-tubulin (**B**, middle panel) antibodies. FM-fetuin-A levels were quantified using α-tubulin as an endogenous reference (**B**, lower panel), with the fetuin-A level without CPP defined as 100% (control). (**C**) HepG2 cells were treated with 100 μg/mL secondary CPP for 0, 6, 12, 24 h. Immunoblots of cell lysates were probed with fetuin-A (**C**, upper panel) and α-tubulin (**C**, middle panel) antibodies. FM-fetuin-A levels were quantified as above (**C**, lower panel), with the fetuin-A level at 0 h defined as 100% (control). Data are shown as mean ± SD from at least three independent experiments. (**D**) Cell lysate from 100 μg/mL secondary CPP-treated HepG2 cells, 40 μg of secondary CPP as a protein level, and 40 μg of bovine fetuin-A were probed with human anti-fetuin-A antibody. (**E**) HepG2 cells were treated with or without 100 μg/mL secondary CPP for 24 h. Cell Lysate and the supernatant from cultured HepG2 cells were probed with an anti-fetuin-A antibody. *P < 0.05 vs. control. Full-length blots/gels (**B**, **C**, **D**, **E**) are presented in Supplementary Fig. X1 (full-size) (**B**, **B′**, **C**, **D**, **D′**, **E**, **E′**), respectively.

We first confirmed formation of synthesized CPP using transmission electron microscopy (TEM). The prepared mixture was gently shaken and incubated at 37 °C for 24 h. At 1.5 h, small spherical-shaped CPP corresponding to primary CPP were observed (Fig. 1A1). After 24 h, these had been replaced by crystalline-like structures with diameters of 100–200 nm, consistent with secondary CPP (Fig. 1A2).

The effect of secondary CPP on the level of FM-fetuin-A in HepG2 cells was investigated by culturing the cells with or without synthetic secondary CPP. The FM-fetuin-A level was increased in a dose-dependent manner by treatment with secondary CPP for 24 h (4.1-fold increase with 100 μg/mL CPP) (Fig. 1B). CPP at 100 μg/mL significantly increased FM-fetuin-A at 12 h, with a further increase at 24 h (Fig. 1C).

To confirm the specificity of anti-fetuin-A antibody, we examined the cross-reactivity between human and bovine fetuin-A (Fig. 1D). This antibody recognized human fetuin-A but not bovine fetuin-A, suggesting the detection of CPP-induced increase of endogenous fetuin-A in HepG2 cells. We also confirmed the secretion of fetuin-A into culture medium. As previously reported^14^, only fully modified fetuin-A was secreted from HepG2 cells. The secretion of FM-fetuin-A significantly increased in the presence of 100 μg/mL secondary CPP (Fig. 1E). On the other hand, each component of secondary CPP (calcium, phosphate, mixture of calcium and phosphate, or fetuin-A) did not change the level of FM-fetuin-A (Supplementary Fig. 1).

### Synthetic secondary CPP have no effect on expression of mRNA for *fetuin-A* in hepatocytes

**Figure 2:**
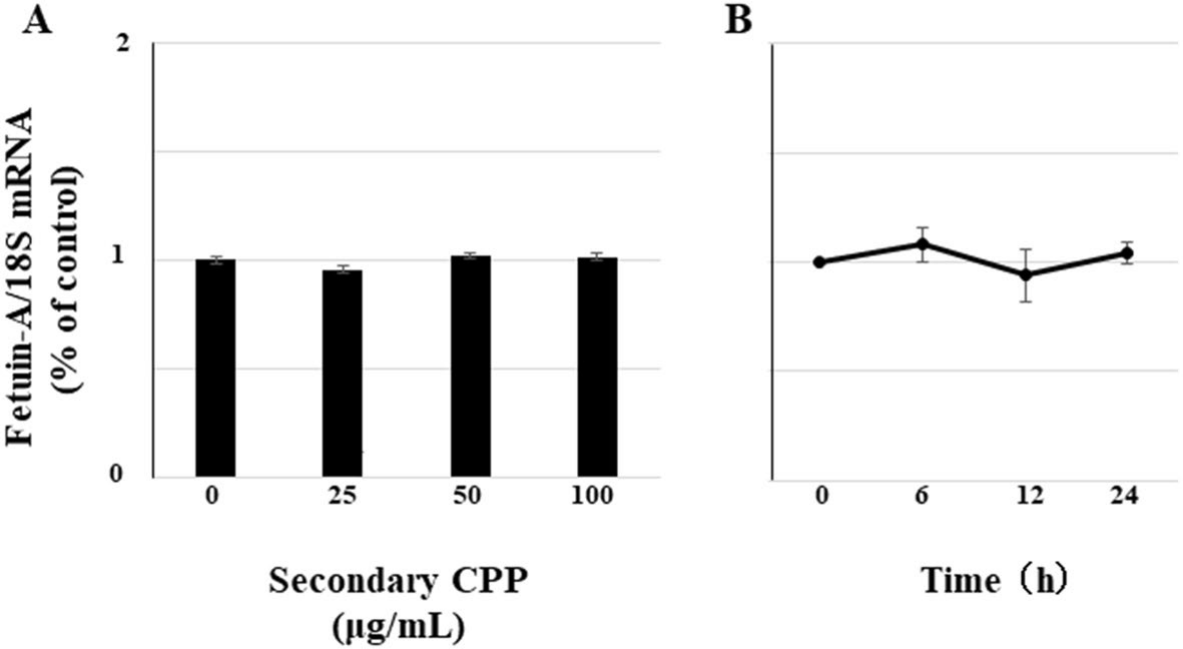
Effects of secondary CPP on expression of *fetuin-A* mRNA in HepG2 cells. (**A**) HepG2 cells were treated with different concentrations (0, 25, 50, and  100 μg/mL) of synthetic secondary CPP for 24 h. (**B**) HepG2 cells were treated with 100 μg/mL secondary CPP for 0, 6, 12, 24 h. Total RNA was isolated from cells and *fetuin-A/ahsg* mRNA expression was determined using quantitative real-time PCR. Levels of *fetuin-A* mRNA were calculated using the comparative cycle threshold method, with 18S ribosomal RNA as the endogenous reference. *Fetuin-A* mRNA levels without secondary CPP (**A**) or at 0 h (**B**) were defined as 100% (control). Data are shown as mean ± SD from at least three independent experiments. *P < 0.05 vs. control.

To examine whether the increased level of FM-fetuin-A originated from CPP-induced expression of mRNA for *fetuin-A* (*ahsg*), real-time quantitative RT-PCR was performed. Unexpectedly, treatment with various concentrations of secondary CPP for 24 h had no effect on the level of *fetuin-A* mRNA, with no change with 100 μg/mL secondary CPP for 24 h (Fig. 2). These findings suggest that secondary CPP induce posttranslational modification of newly-synthesized native fetuin-A without a change of *fetuin-A* transcription.

### Localization of secondary CPP in HepG2 cells

**Figure 3:**
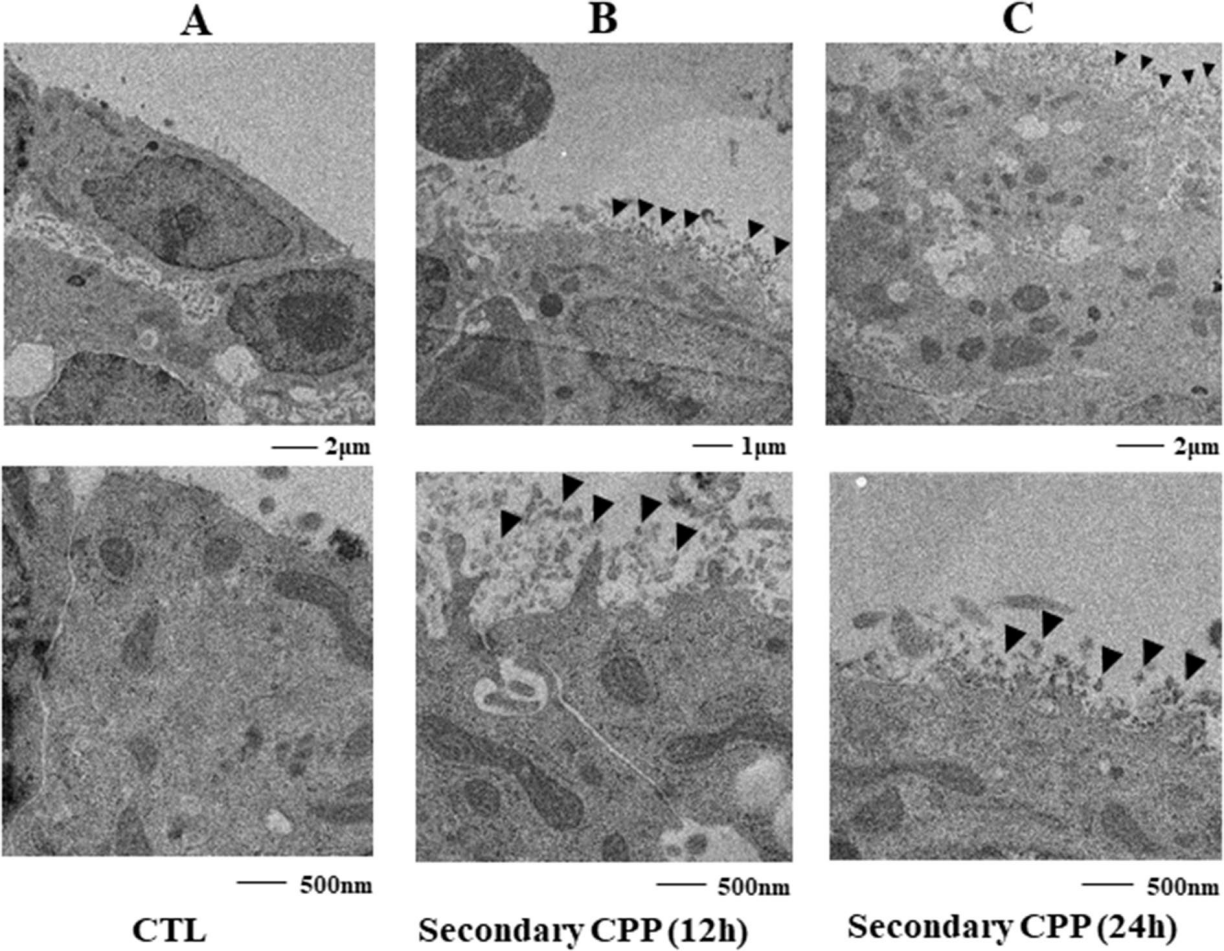
Localization of secondary CPP in HepG2 cells. HepG2 cells without incubation with secondary CPP (**A**) and 12 h (**B**) and 24 h (**C**) after addition of 100 μg/mL secondary CPP. TEM imaging showed extracellular crystalline-like structures (arrowhead) around treated HepG2 cells (**B**, **C**), whereas no such structure was present in control cells (**A**).

Synthetic CPP-induced inflammation or calcification has been suggested to be mediated by cellular uptake of secondary CPP. After exposure of phagocytic Kupffer cells, macrophages and VSMCs to secondary CPP, TEM imaging showed an intracellular crystalline-like structure, suggesting active transport of secondary CPP into Kupffer cells^13^, macrophages^6^, and VSMCs^7^. Therefore, we tried to visualize the localization of synthetic CPP in the hepatocyte culture. At 12 h, crystalline-like structures of added secondary CPP were observed around HepG2 cells (Fig. 3B), but there were no intracellular structures. After 24 h, these structures were still absent, although CPP-like structures continued to surround the cells (Fig. 3C). These findings suggest that secondary CPP may transduce signals into cells without CPP uptake.

### Deglycosylation of secondary CPP-induced FM-fetuin-A by O- and N-glycosidases and secondary CPP-induced serine-phosphorylation of FM-fetuin-A

**Figure 4:**
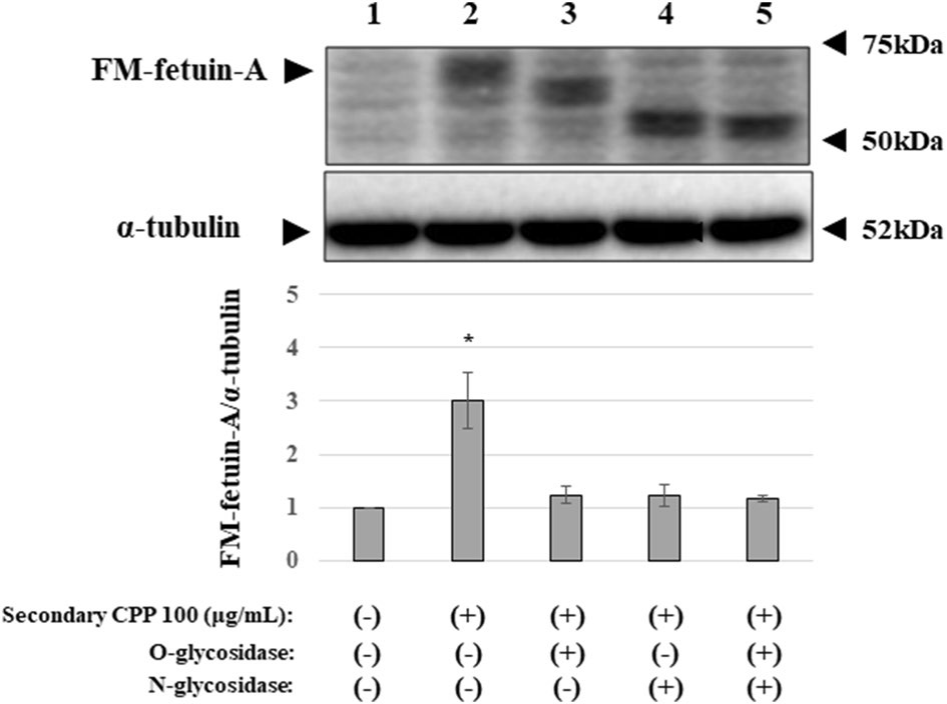
Deglycosylation of secondary CPP-induced FM-fetuin-A by O- and N-glycosidases. Secondary CPP-treated cell lysates were denatured at 100 °C for 10 min and subsequently incubated with O-glycosidase and/or N-glycosidase F (PNGase F) at 37 °C for 1–2 h. Immunoblots of enzyme-treated samples were probed with fetuin-A (upper panel) and α-tubulin (lower panel) antibodies. FM-fetuin-A levels were quantified using α-tubulin as an endogenous reference, with the fetuin-A level in the absence of CPP without O-glycosidase and N-glycosidase F defined as 100% (control). Data are shown as mean ± SD from at least three independent experiments. *P < 0.05 vs. control. Full-length blots/gels are presented in Supplementary Fig. X4 (full-size).

A previous study showed posttranslational processing of fetuin-A in HepG2 cells, including O- and/or N-glycosylation^14^, and the MW of fetuin-A in an immunoblot depends on the degrees of these changes. To confirm glycosylation of FM-fetuin-A, secondary CPP-treated cell lysates were treated with O- and/or N-glycosidases. O-glycosidase treatment caused a slightly lower shift of the 60 kDa band of FM-fetuin-A (Fig. 4, lane 3), and this band shifted to a much lower position in the presence of N-glycosidase alone (Fig. 4, lane 4) or with O-glycosidase (Fig. 4, lane 5). These findings show that posttranslational modifications of fetuin-A included various glycosylations.

In addition, fetuin-A is modified by phosphorylation on serine residues^1–3^. Secondary CPP induced serine-phosphorylations of FM-fetuin-A in a dose dependent manner (Supplementary Fig. 2). However, serine-phosphorylations of FM-fetuin-A had little effect on MW of FM-fetuin-A.

### Effect of intracellular blockade of protein transport on the secondary CPP-induced FM-fetuin-A

**Figure 5:**
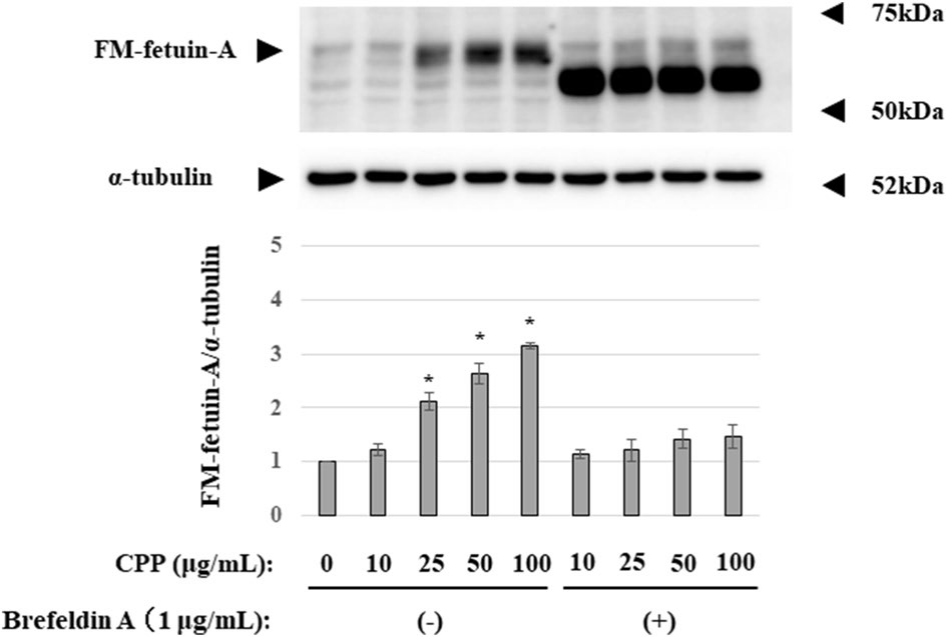
Effect of brefeldin A on secondary CPP-induced FM-fetuin-A. HepG2 cells were pre-treated for 30 min with or without 1 mg/mL brefeldin A and subsequently incubated in the absence or presence of the indicated concentrations of secondary CPP for 24 h. Immunoblots of cell lysates were probed with fetuin-A (upper panel) and α-tubulin (lower panel) antibodies. FM-fetuin-A levels were quantified using α-tubulin as an endogenous reference, with the fetuin-A level in the absence of CPP without brefeldin A defined as 100% (control). Data are shown as mean ± SD from at least three independent experiments. *P < 0.05 vs. control. Full-length blots/gels are presented in Supplementary Fig. X5 (full-size).

To investigate whether secondary CPP increase FM-fetuin-A through promotion of posttranslational modifications, we used brefeldin A to inhibit intracellular protein transport from the endoplasmic reticulum (ER) to the Golgi apparatus^14,15^. Preincubation with brefeldin A completely inhibited the CPP-induced increase in FM-fetuin-A (Fig. 5). This suggests that secondary CPP may promote processing of native fetuin-A, resulting in an increased FM-fetuin-A level without alteration of mRNA expression.

### Effect of intracellular calcium chelation on secondary CPP-induced FM-fetuin-A

**Figure 6:**
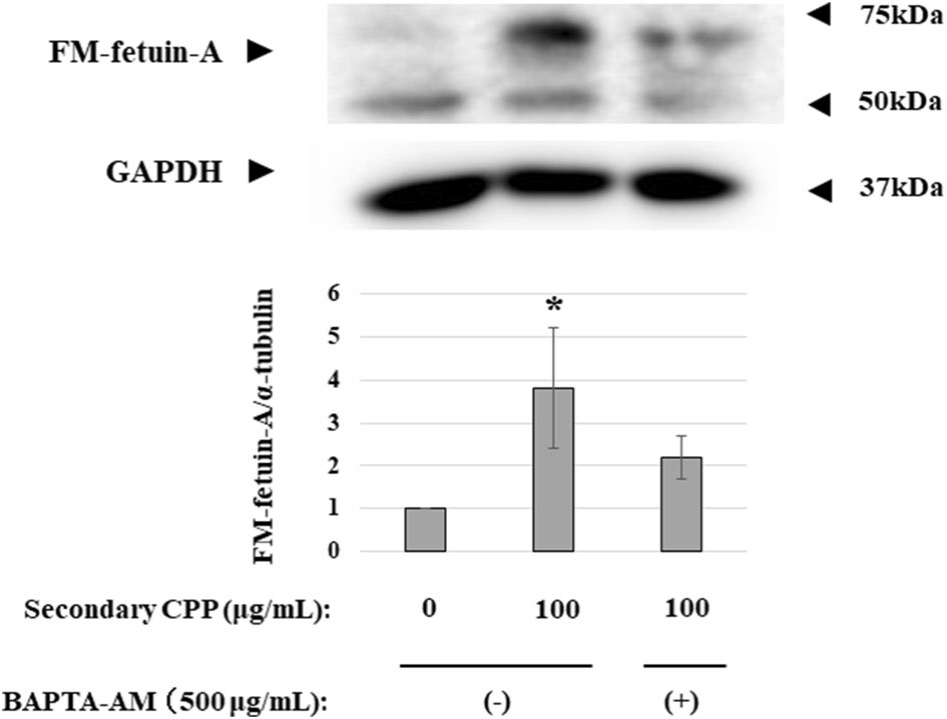
Effect of BAPTA-AM on secondary CPP-induced FM-fetuin-A. HepG2 cells were cultured with 100 μg/mL secondary CPP in the absence or presence of 500 μg/mL of BAPTA-AM for 24 h. Immunoblots of cell lysates were probed with fetuin-A (upper panel) and GAPDH (lower panel) antibodies. FM-fetuin-A levels were quantified using GAPDH as an endogenous reference, with the fetuin-A level in the absence of CPP without BAPTA-AM as 100% (control). Data are shown as mean ± SD from at least three independent experiments. *P < 0.05 vs. control. Full-length blots/gels are presented in Supplementary Fig. X6 (full size).

Intracellular calcium plays a critical role in vesicle fusion and vesicle formation, which lead to protein transport. Since an increase in intracellular calcium can induce vascular calcification^7^, we used 1,2-bis (2-aminophenoxy)ethane-*N*, *N*, *N′*, *N′*-tetraacetic acid–tetrakis (acetoxymethyl ester) (BAPTA-AM) as an intracellular calcium chelator in HepG2 cells treated with 100 μg/mL CPP. Treatment with BAPTA-AM inhibited the secondary CPP-induced increase in FM-fetuin-A (Fig. 6). This suggests that secondary CPP may partially promote formation of FM-fetuin-A in a calcium-dependent manner.

## Discussion

In this study, we found that synthetic secondary CPP induce formation of FM-fetuin-A without a change in *fetuin-A* mRNA expression in HepG2 cells. Native fetuin-A undergoes posttranslational modifications, including O- and N-glycosylations, to form FM-fetuin-A. Blockade of protein transport from the ER to the Golgi apparatus or intracellular calcium chelation inhibited secondary CPP-induced FM-fetuin-A formation. These findings suggest that secondary CPP accelerate posttranslational processing in HepG2 cells.

We originally expected to see upregulation of FM-fetuin-A through secondary CPP-induced expression of mRNA for *fetuin-A/ahsg*. Since this was not found (Fig. 2), we next focused on degradation of FM-fetuin-A. However, a lysosome inhibitor (chloroquine) (Supplementary Fig. 3) and a ubiquitin–proteasome inhibitor (lactacystin) (Supplementary Fig. 4) had no effect on the FM-fetuin-A level, suggesting no involvement of secondary CPP in degradation of FM-fetuin-A. We also considered the possibility that bovine fetuin-A derived from synthetic secondary CPP might have been detected in immunoblotting. However, since human fetuin-A antibody did not recognize bovine fetuin-A (Fig. 1D), we concluded that the observed increase was of intrinsic FM-fetuin-A in HepG2 cells, rather than due to uptake of fetuin-A that had dissociated from synthetic secondary CPP.

We next focused on posttranslational processing of fetuin-A. Among various modifications, the degree of O- or N-glycosylation affects the MW of fetuin-A (Fig. 4). Secondary CPP had no effect on the native fetuin-A level because there was no change in the mRNA level. Generally, newly synthesized proteins go through the ER, the intermediate compartment or vesicular-tubular clusters and the Golgi complex in a sequential manner^15^. Traffic among these membrane-bounded compartments are mediated by coated vesicular proteins, COP I and COP II^15^. During this process, newly synthesized proteins undergo changes such as glycosylation. Brefeldin A completely abolished the secondary CPP-induced increase in FM-fetuin-A (Fig. 5). Since brefeldin A inactivates ARF, a component of COP I, resulting in inhibition of transport from the ER to the Golgi complex, secondary CPP may accelerate the transport process accompanied by O- and N-glycosylation. Intracellular calcium chelation by BAPTA-AM also inhibited the secondary CPP-induced increase in FM-fetuin-A. Calcium can regulate membrane fusion reactions and is required for maintaining the integrity of the membrane coat. Thus, calcium chelators ‘uncoat’ vesicle membranes^16^. We speculate that secondary CPP modulate native fetuin-A transport from the ER to the Golgi complex, in part in a calcium-dependent manner.

Previous studies suggest that uptake of secondary CPP into cells could trigger pathological actions based on formation of CPP-like structures inside the cells. However, we were unable to find such structures inside HepG2 cells, although CPP-like structures surrounded the cells. Apart from secondary CPP, calcium phosphate precipitates can enter cells by clathrin-dependent endocytosis^17^. This phenomenon also allows DNA transfection. To examine whether internalization of CPP into HepG2 cells is necessary, we used dynasore to inhibit clathrin-dependent coated vesicle formation^17^. Treatment with dynasore did not affect the CPP-induced FM-fetuin-A level (Supplementary Fig. 5), which suggests that it is unlikely that the change in FM-fetuin-A is mediated by uptake or internalization of secondary CPP.

Finally, we tested the hypothesis that CPP transduce extracellular signals into cells through toll-like receptor 4 (TLR4). Fatty acids and fetuin-A can exacerbate insulin resistance^18^, with fetuin-A functioning as an adaptor protein between fatty acids and TLR4^19^. Therefore, we used TAK-242 to inhibit interactions between TLR4 and its intracellular adaptor molecules. However, TAK-242 had no effects on secondary CPP-induced FM-fetuin-A (Supplementary Fig. 6). We also examined if secondary CPP could bind to TLR4 and produce subsequent signal transduction in HEK 293 cells stably transfected with TLR4, its intracellular related molecules, and a reporter construct. This system supported lipopolysaccharide-stimulated TLR4-induced signal transduction, whereas CPP did not have this effect (Supplementary Fig. 7). Therefore, we conclude that secondary CPP do not change the FM-fetuin-A level through TLR4.

There are several limitations in this study. First, we examined the effects of synthetic secondary CPP on FM-fetuin-A. Therefore, it is unclear whether primary CPP could act on the level of FM-fetuin-A. Second, it was not completely denied that internalized secondary CPP and/or its fragment could influence FM-fetuin-A, although CPP-like structures were not found inside the cells in TEM images and clathrin-mediated endocytosis was not involved in CPP-induced increase in FM-fetuin-A. It is hypothesized that protein-mineral complexes such as CPP are endocytosed and interact with various vesicles including secretory proteins^20^. Subsequent formation and release of extracellular vesicles are thought to play crucial role in biomineralization^20^. Beyond calcified tissues, careful examination about the movement and transformation of CPP is further required. Third, since fetuin-A is heavily glycosylated, it may be possible that CPP could accelerate glycosylations independent of posttranslational processing, resulting in increased levels of FM-fetuin-A. However, CPP also enhanced serine phosphorylations of FM-fetuin-A and its subsequent secretion into culture medium. Considering these findings, CPP could promote overall posttranslational modifications.

The inhibitory effect of fetuin-A on ectopic calcification is clear, and this process requires formation of CPP. Such CPP may be cleared by the reticuloendothelial system in mice^13^, but the fate of an unmanageable overload of CPP is unclear. For example, CPP are detectable in patients with advanced CKD, especially if these patients are undergoing dialysis^11,12^. Given the inverse relationship of decreased fetuin-A with increased CPP, it is hypothesized that fetuin-A forms CPP that subsequently stimulate fetuin-A production against fetuin-A consumption under calcification stress in advanced CKD. Our result showing a secondary CPP-induced increase in FM-fetuin-A in HepG2 cells also suggests a positive feedback loop between secondary CPP and fetuin-A. Further studies are necessary to confirm whether secondary CPP can upregulate hepatic fetuin-A secretion in vivo in humans.

## Materials and methods

### Synthesis of CPP

CPP were synthesized as previously reported^6,21^. Briefly, a CPP solution containing 1 mg/mL bovine fetuin-A (Sigma-Aldrich, St. Louis, MO, USA), 10 mM CaCl_2_, 6 mM Na_2_HPO_4_, 140 mM NaCl, and 50 mM Tris–HCL (pH 7.4) was gently shaken and incubated at 37 °C for at least 24 h. Synthetic CPP were separated using a spin-filtered membrane cartridge with a 300 kDa cut-off (Sartorius AG, Göttingen, Germany). CPP concentrations were determined by bicinchoninic acid (BCA) assay as a protein level. The ionic calcium content of secondary CPP was also measured by Calcium Assay Kit (Cat No. ab102505, abcam, Cambridge, UK). One hundred μg/mL of secondary CPP as a protein concentration included 45 μg/mL of ionic calcium (Supplementary Fig. 8). Synthetic secondary CPP were used for experiments from 24 to 168 h after the first mixture of the solution.

### TEM

The CPP solution was incubated at 37 °C for 1.5 h, 2.5 h, and 24 h, as described above. To confirm synthesis of CPP, primary and secondary CPP were visualized by TEM. The incubated CPP solution was applied to Formvar (polyvinyl formal)-coated copper grids (Veco, Eerbeek, Netherlands). After the grids were dried at room temperature, CPP were negatively stained with uranyl acetate and visualized using a H-7500 electron microscope (Hitachi, Tokyo, Japan) at 80 kV.

### Cell culture

A human hepatoma cell line (HepG2) was purchased from American Type Culture Collection (ATCC, Manassas, VA, USA) and cultured in Dulbecco’s modified Eagle medium (DMEM; Nacalai Tesque, Kyoto, Japan) supplemented with 10% fetal bovine serum (Sigma-Aldrich), 100 U/mL penicillin and 100 µg/mL streptomycin in a humidified atmosphere containing 5% CO_2_ at 37 °C^21^. The medium was replaced every 2 days. After the cultures reached confluence, cells were serum-starved overnight and subsequently incubated for 0, 6, 12, or 24 h with different concentrations (0, 1, 5, 10, 25, 50, 100, and 200 μg/mL) of synthetic secondary CPP.

### Immunoblotting

HepG2 cells were washed twice with ice-cold buffer (137 mM NaCl, 1 mM MgCl_2_, 1 mM CaCl_2_, 0.1 mM Na_2_VO_4_, and 20 mM Tris–HCl, pH 7.6) and then lysed in the same buffer supplemented with 1% Nonidet P-40, 10% glycerol, 2 mM EDTA, 10 mM sodium pyrophosphate, 10 mM NaF, 2 mM Na_2_VO_4_, 2 mM phenylmethylsulfonyl fluoride, and 8 µg/mL leupeptin. Protein concentrations were determined using BCA protein assays (Thermo Scientific, MI, USA). Cell lysates (40 µg protein per lane) were subjected to SDS-PAGE, transferred to nitrocellulose membranes (GE Healthcare Life Sciences, Buckinghamshire, UK), and incubated with antibodies against human fetuin-A (Cat No. AF1184, R&D Systems, Minneapolis, MN, USA), α-tubulin and GAPDH (both Cell Signalling Technology, Danvers, MA, USA). Protein bands were identified using ECL (GE Healthcare Life Sciences) and quantified using a densitometer (Lumino Shot 400Jr) (Takara Bio Inc, Shiga, Japan)^22,23^.

### RNA isolation and real-time quantitative RT-PCR

RNA was extracted form HepG2 cells using TRIzol reagent (Invitrogen, Waltham, MA, USA), and 2 µg was used to synthesize cDNA by reverse transcription using a High Capacity cDNA Reverse Transcription Kit (Applied Biosystems, Foster City, CA, USA). Primers for human *fetuin-A (ahsg)* (Hs00155659) and 18S ribosomal RNA (4319413E) were purchased from Applied Biosystems (TaqMan Gene Expression Assays). Expression of human *fetuin-A* and 18S ribosomal RNA were quantified using TaqMan Real-Time PCR (Applied Biosystems). A cycle threshold (Ct) value was determined, and each mRNA level was quantified using the comparative Ct method with 18S ribosomal RNA as the endogenous control^22,23^.

### CPP localization

HepG2 cells were cultured with or without 100 μg/mL CPP for the indicated periods. To assess the localization of synthetic secondary CPP, HepG2 cells were fixed with 2.5% glutaraldehyde and 2% paraformaldehyde in 0.1 M phosphate buffer for 30 min, post-fixed with 1% osmium tetroxide for 30 min at 4 °C, dehydrated, and embedded in Quetol-812. Ultrathin sections were obtained at a thickness of 60–70 nm and applied to Formvar. Specimens were negatively stained with uranyl acetate and lead citrate, and subsequently captured using a H-7500 electron microscope at 80 kV.

### Posttranslational modifications

Fetuin-A undergoes various posttranslational modifications^14^, and FM-fetuin-A migrates at about 60 kDa in an immunoblot, which is much higher than the deduced MW based on the amino acid sequence^22,23^. FM-fetuin-A has two N-linked and three O-linked glycosylation sites. To confirm these posttranslational modifications, enzymatic deglycosylation was performed in CPP-treated lysates. Each lysate was denatured at 100 °C for 10 min and then incubated with O-glycosidase or N-glycosidase F (PNGase F) (both New England Biolabs Japan Inc., Tokyo, Japan) at 37 °C for 1–2 h. Brefeldin A (Cosmo Bio Japan Inc., Tokyo, Japan) was used to inhibit transport of proteins from the ER to the Golgi apparatus. After pretreatment for 30 min with or without 1 mg/mL brefeldin A, cells were incubated with the indicated concentrations of secondary CPP for 24 h. Since intracellular calcium plays a critical role in regulating protein trafficking via membrane fusion reactions, BAPTA-AM (Cosmo Bio Japan Inc.) was used as an intracellular calcium chelating agent. Cells were cultured with or without 100 μg/mL secondary CPP and 500 μg/mL BAPTA-AM for 24 h. Samples treated with glycosidases, brefeldin A and BAPTA-AM were analyzed by immunoblotting, as described above.

### Statistical analysis

Each experiment was repeated at least thrice. Data are presented as means ± standard deviation (SD). Differences among more than three groups were analysed using ANOVA followed by a Dunnett post hoc test, using StatView (SAS Institute Inc., Cary, NC, USA) on a Windows PC^22,23^. The significance level was set at *P* < 0.05.

## Supplementary Information

Supplementary Figures.

## Abbreviations

CPP: Calciprotein particles
MW: Molecular weight
FM-fetuin-A: Fully-modified fetuin-A
CKD: Chronic kidney disease
BAPTA-AM: 1,2-Bis (2-aminophenoxy)ethane-*N*, *N*, *N′*, *N′*-tetraacetic acid-tetrakis (acetoxymethyl ester)
VSMCs: Vascular smooth muscle cells
TEM: Transmission electron microscopy
