# Supplementary material for: Effects of fetuin-A-containing calciprotein particles on posttranslational modifications of fetuin-A in HepG2 cells
Source: Sci Rep. 2021 Apr 5;11:7486. doi: 10.1038/s41598-021-86881-0 (PMC8021573; doi:10.1038/s41598-021-86881-0)

Supplementary Figures

Effects of fetuin-A-containing calciprotein particles on posttranslational modifications of fetuin-A in HepG2 cells

Hideki Uedono[1], Katsuhito Mori[2], Akinobu Ochi[1], Shinya Nakatani[1], Yuya Miki[1], Akihiro Tsuda[1], Tomoaki Morioka[1], Yuki Nagata[3] Yasuo Imanishi[1], Tetsuo Shoji[3,4]
Masaaki Inaba[1,2], Masanori Emoto[1,2]

[1]Department of Metabolism, Endocrinology and Molecular Medicine, [2]Department of Nephrology, [3]Department of Vascular Medicine, [4] Vascular Science Center for Translational Research, Osaka City University Graduate School of Medicine, Osaka, Japan

Supplementary Figure 1

The effects of calcium, phosphate, mixture of calcium and phosphate or fetuin-A on FM-fetuin-A in HepG2 cells

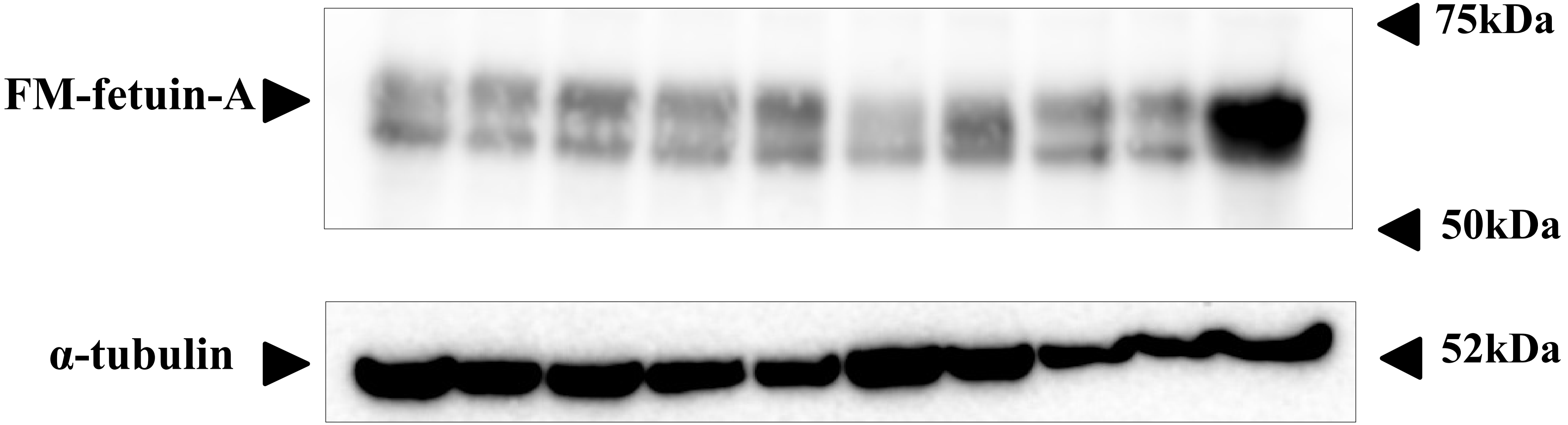

	Control	Calcium		Phosphate		Mixture of calcium and phosphate		Fetuin-A		Secondary CPP
Calcium (mM)	(-)	0.5	3.0	(-)	(-)	1.6	3.2	(-)	(-)	(-)
Phosphate (mM)	(-)	(-)	(-)	0.5	6.0	1.0	2.0	(-)	(-)	(-)
Fetuin-A (µg/mL)	(-)	(-)	(-)	(-)	(-)	(-)	(-)	100	300	(-)
Secondary CPP (µg/mL)	(-)	(-)	(-)	(-)	(-)	(-)	(-)	(-)	(-)	100

Supplementary Figure 2

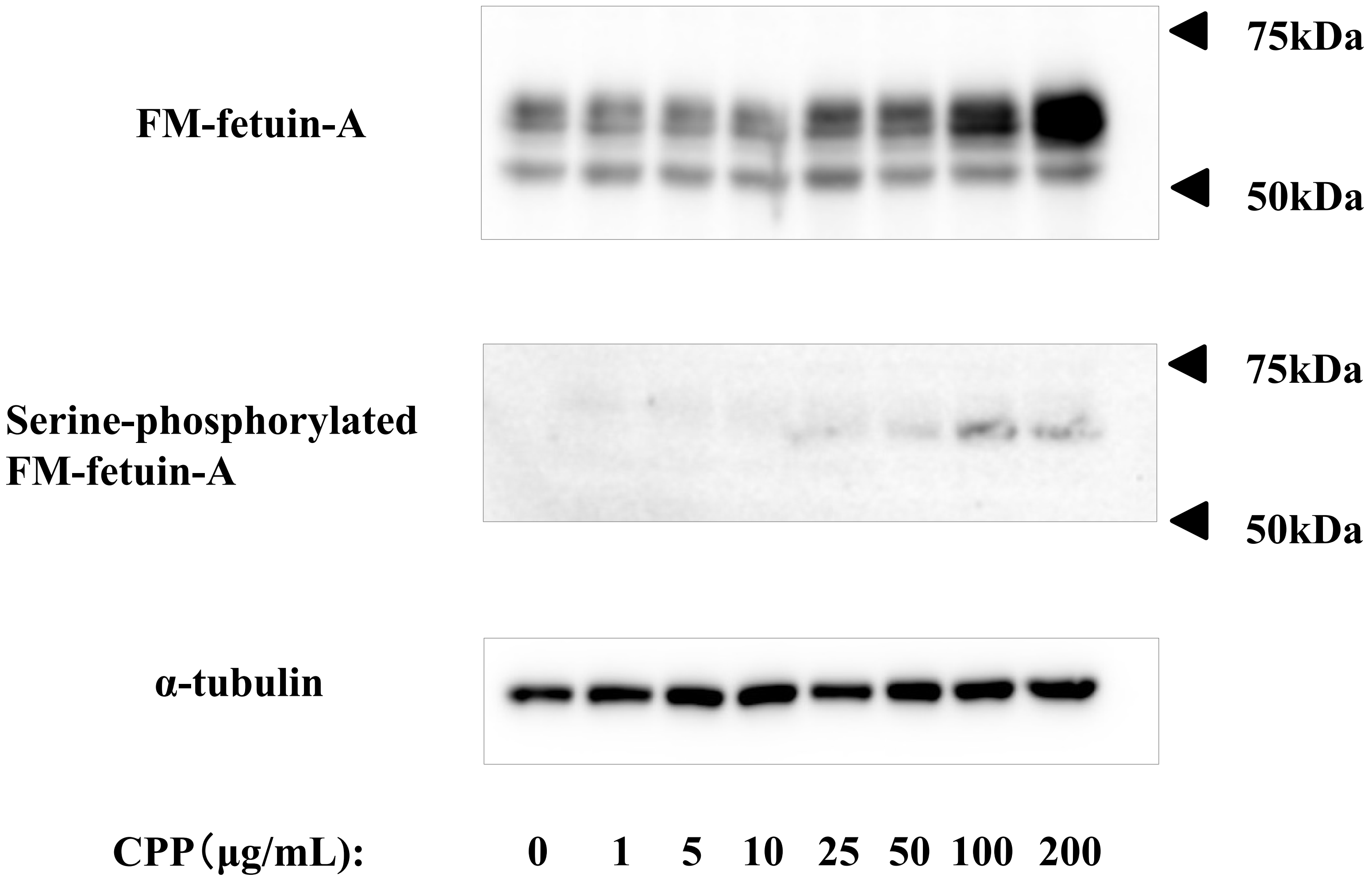

Anti-phosphorylated serine antibody (Cat No. 4139, Cell Signaling Technology, MA, USA)

Supplementary Figure 3

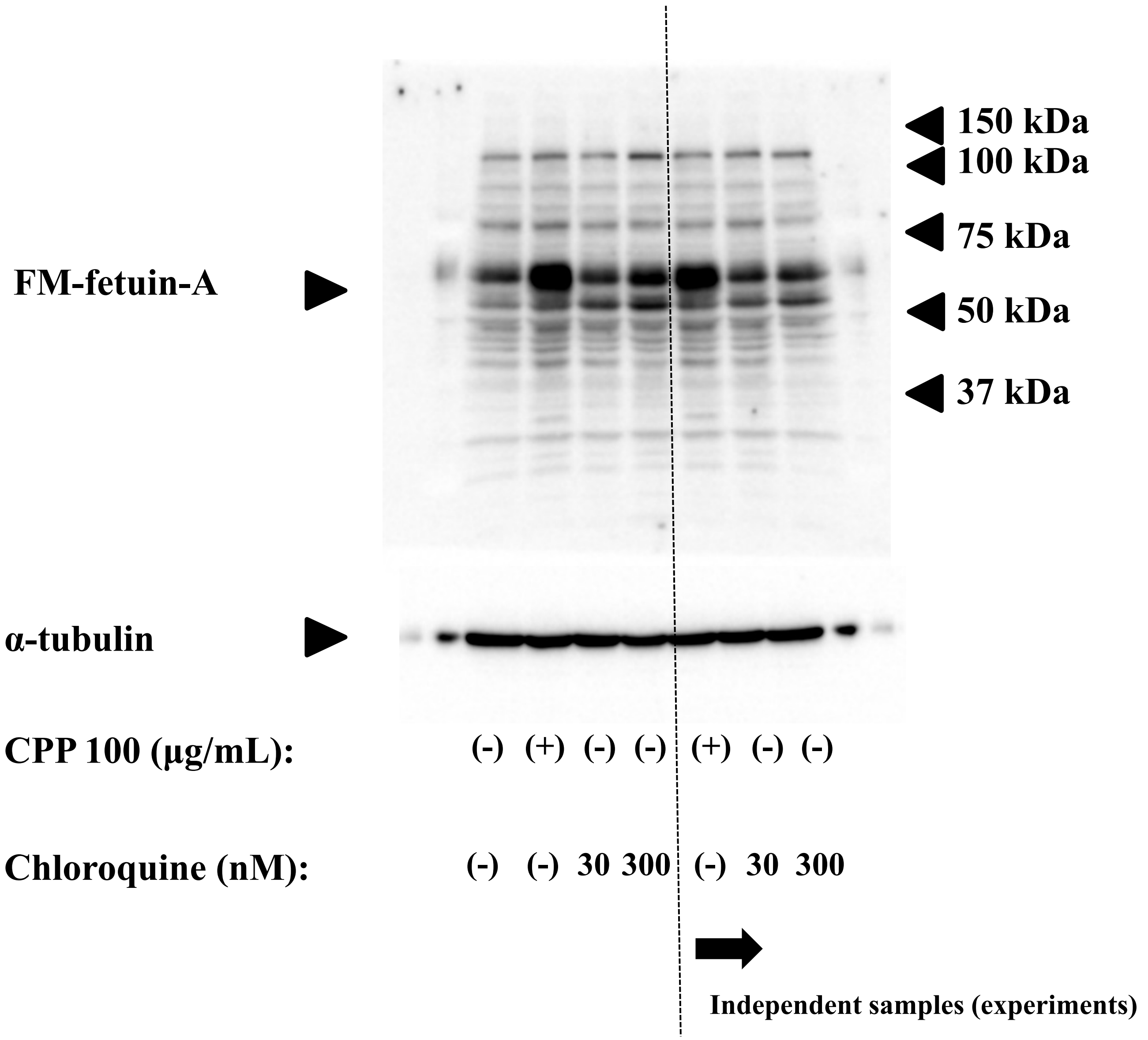

Supplementary Figure 4

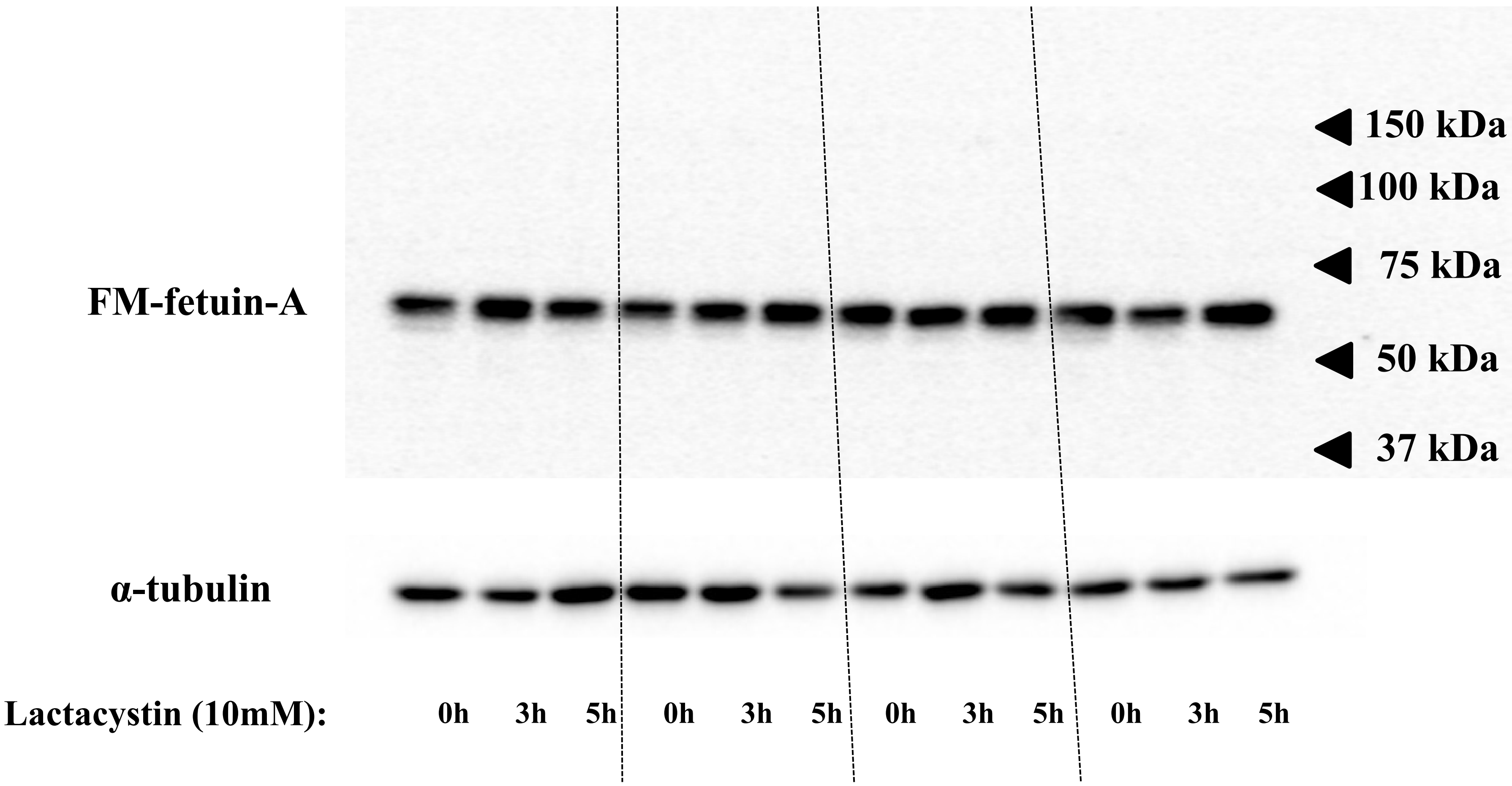

This immunoblot consists of four independent experiments (samples).

Supplementary Figure 5

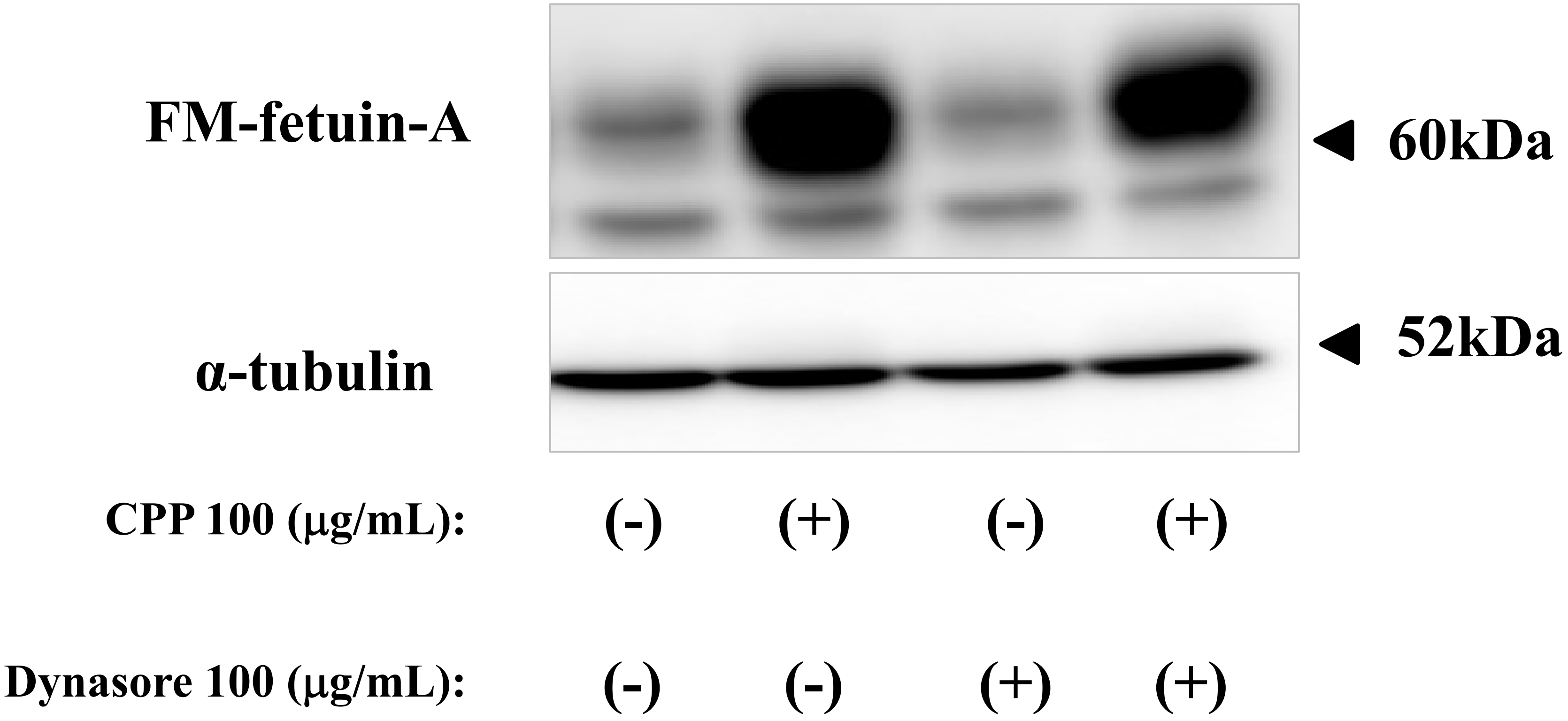

Supplementary Figure 6

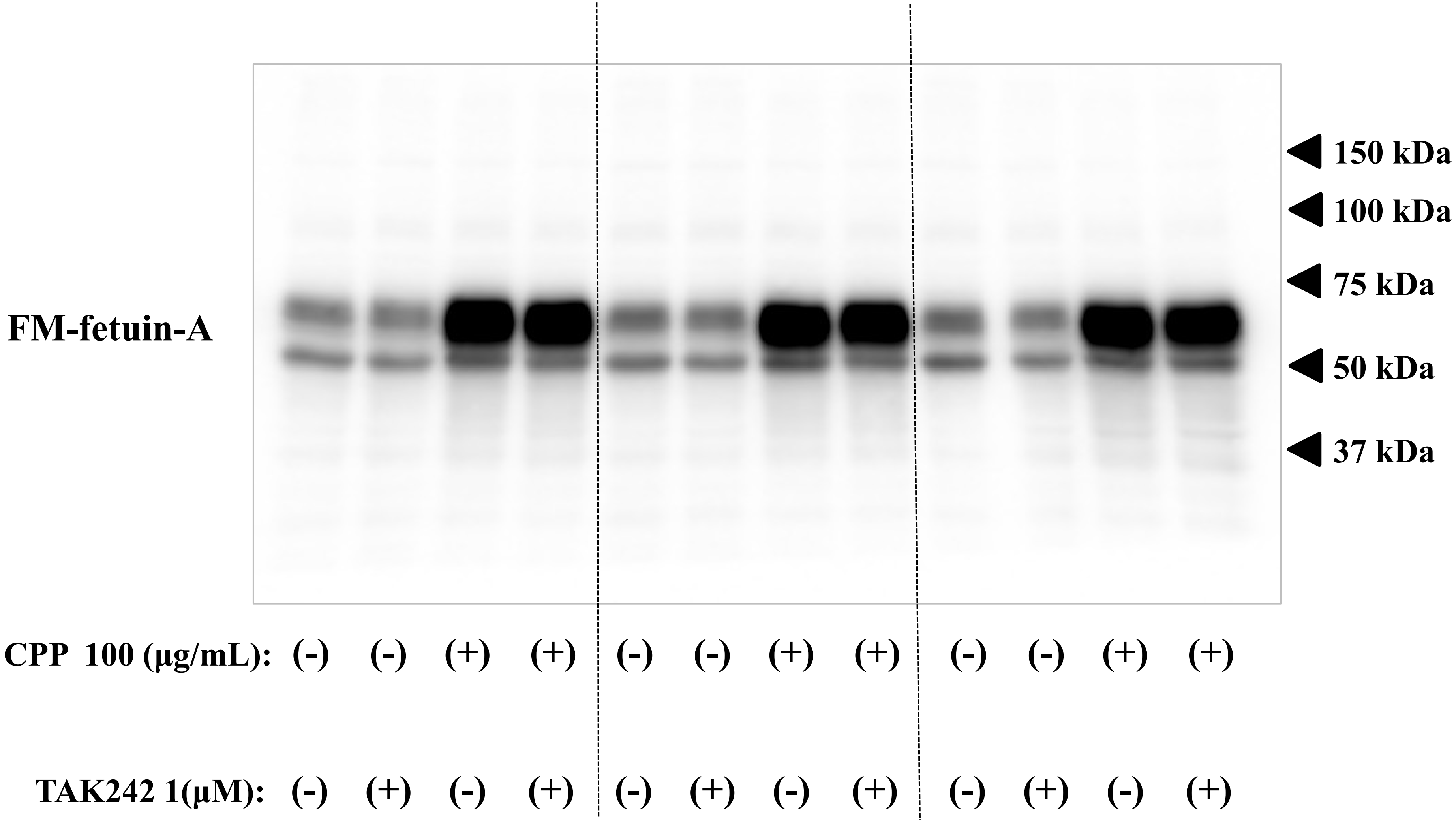

This immunoblot consists of three independent experiments (samples).

Supplementary Figure 7

Effects of secondary CPP on SEAP secretion in HEK 293 cells stably transfected TLR4/MD-2/CD14/NFκB/SEAP reporter construct

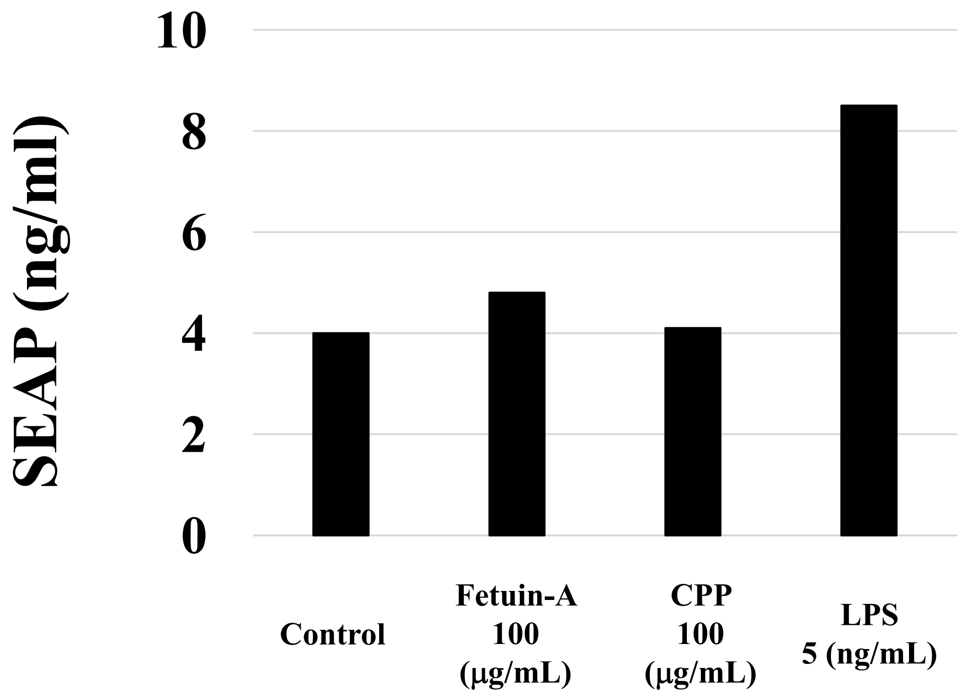

SEAP: secretable alkaline phosphatase

Supplementary Figure 8

The relationship between protein concentration and ionic calcium concentration in secondary CPP

Secondary CPP (μg/mL)	0	10	25	50	100	200
Ionic calcium content (mg/dL)	0	0.81	1.86	3.21	4.50	7.26
Ionic calcium content (μg/mL)	0	8.1	18.6	32.1	45.0	72.6

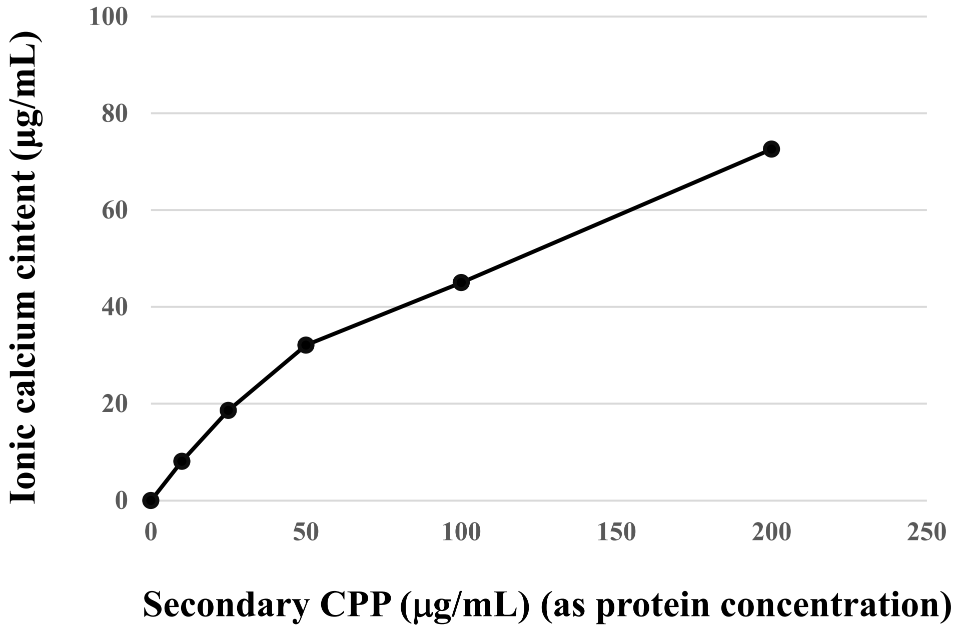

Calcium content of secondary CPP was determined by the Calcium Assay Kit (Cat No. ab102505, Cambridge, UK).

Supplementary Figure X1 (full-size)

(B) (full-length blots)
FM-fetuin-A

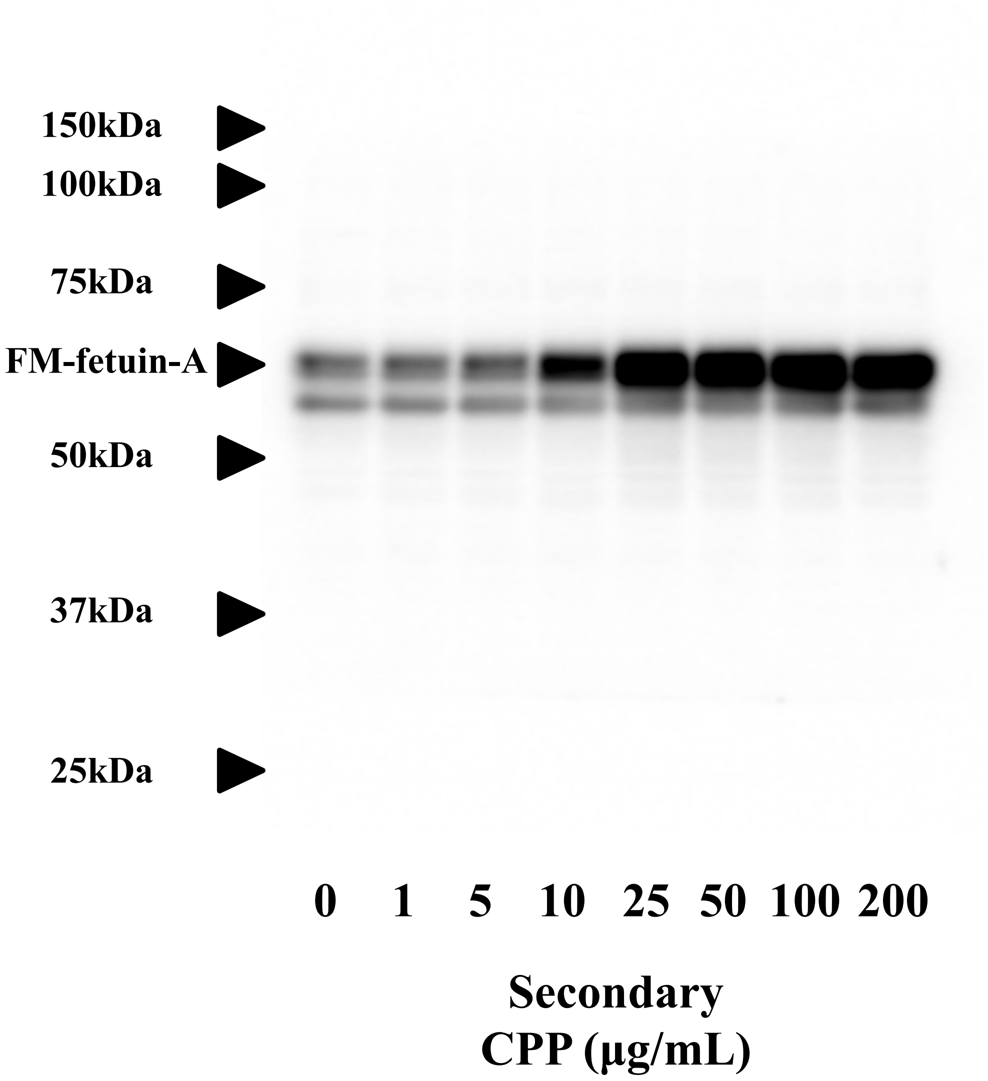

(B') (high-contrast image to make membrane edges visible using same membrane)
FM-fetuin-A

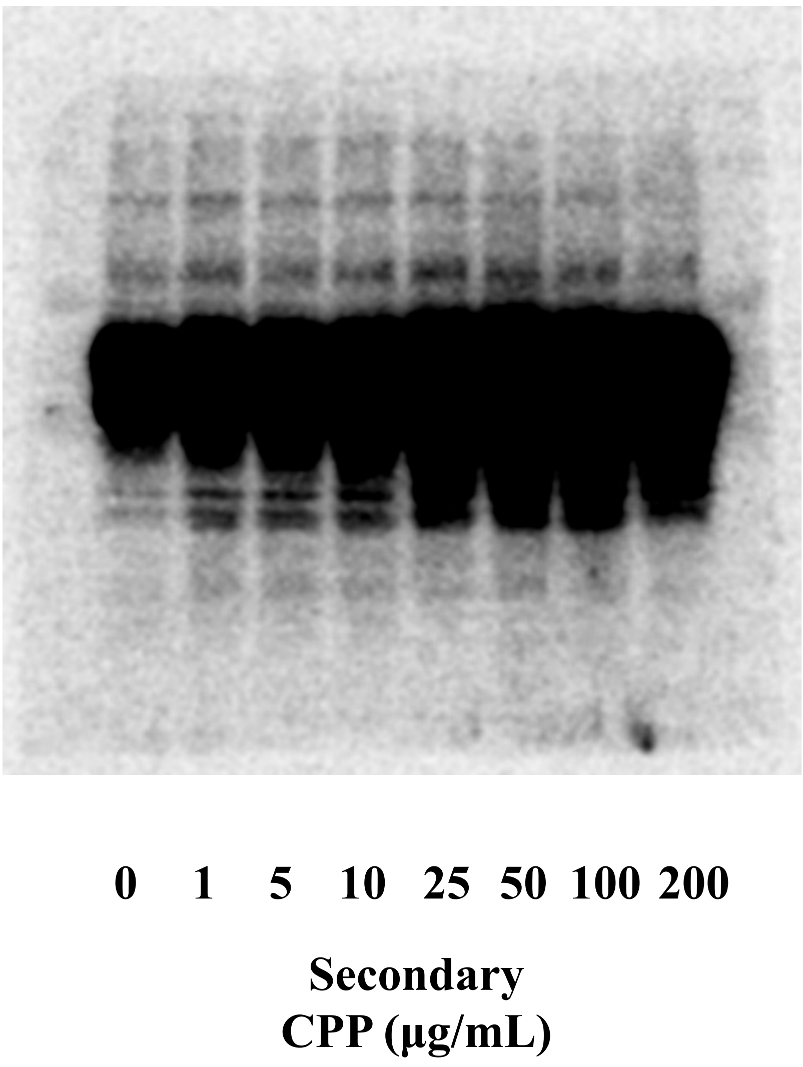

Supplementary Figure X1 (full-size)

(B) (full-length blots)
α-tubulin

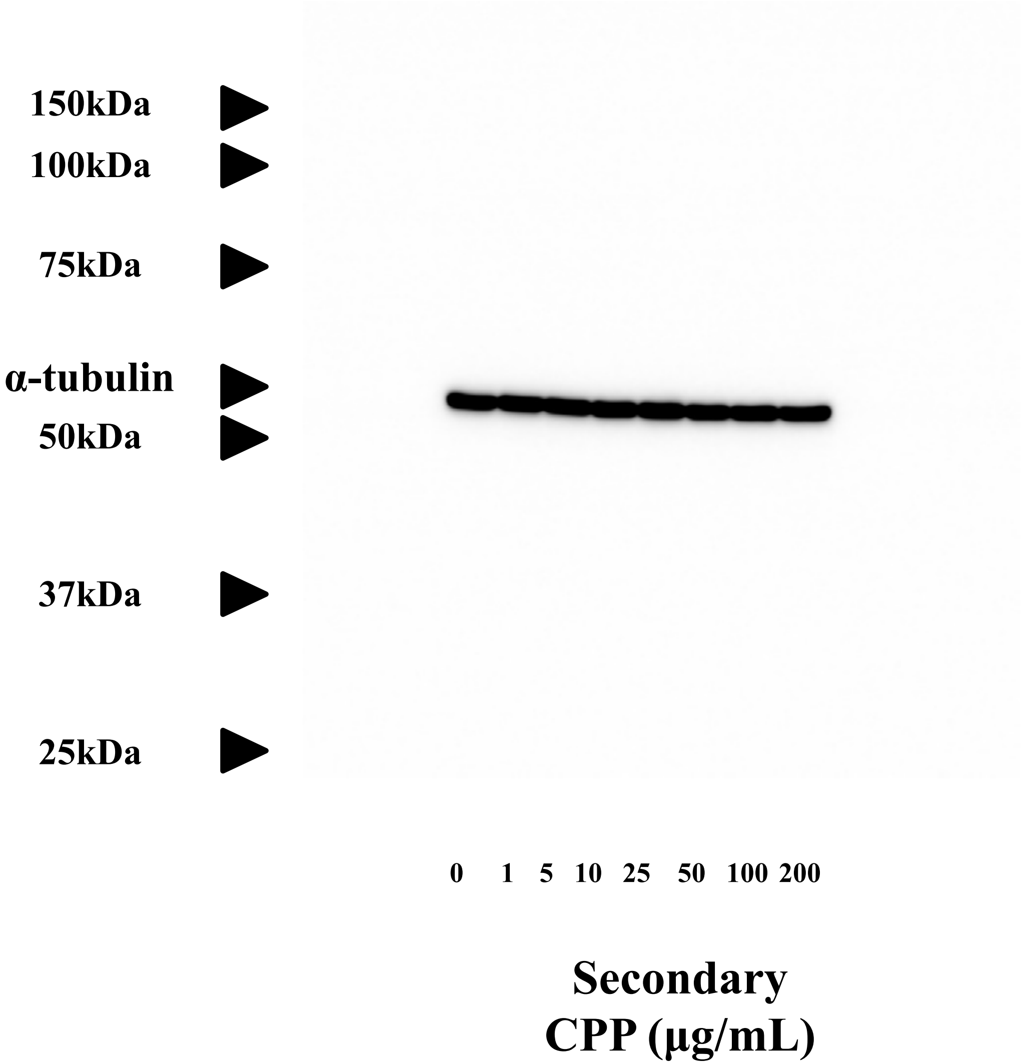

(B') (full-length blots with enhanced contrast without any clopping)
α-tubulin

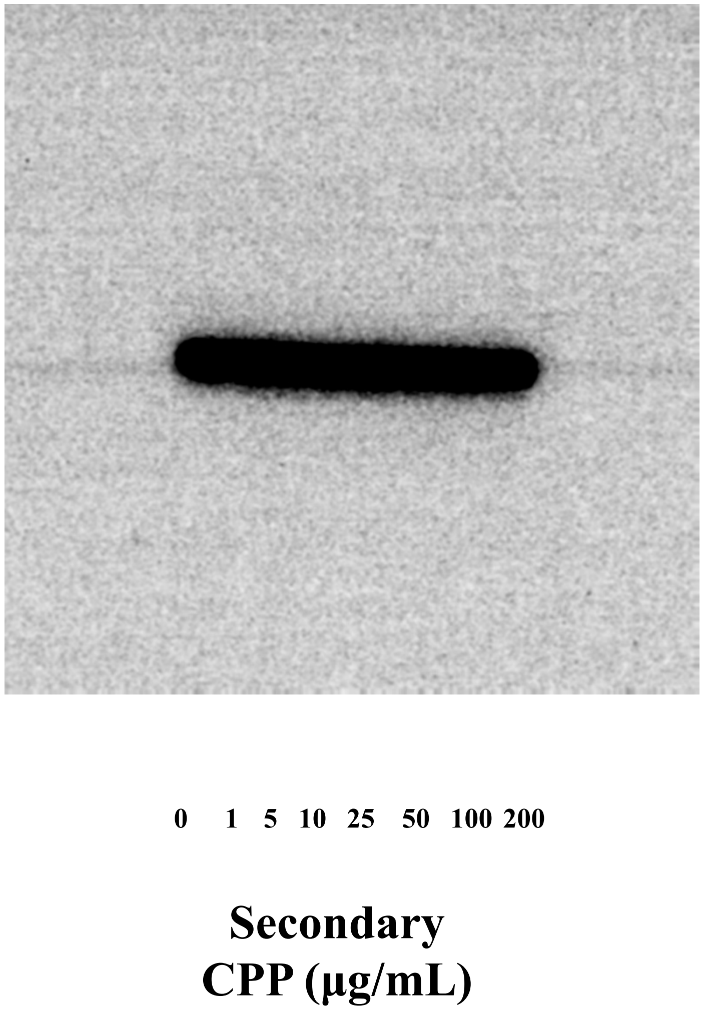

Supplementary Figure X1 (full-size)

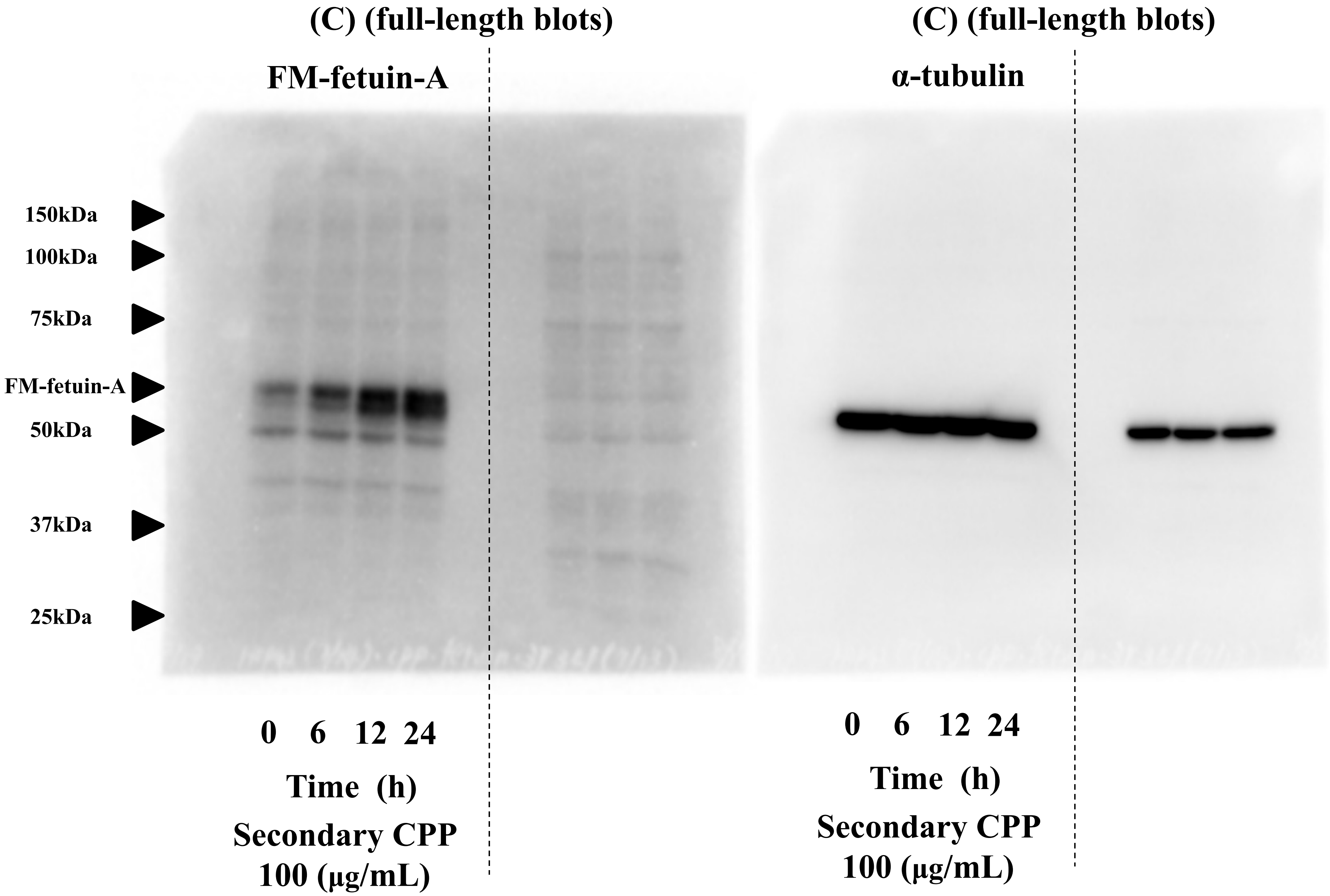

Supplementary Figure X1 (full-size)

(D) (full-length blots)

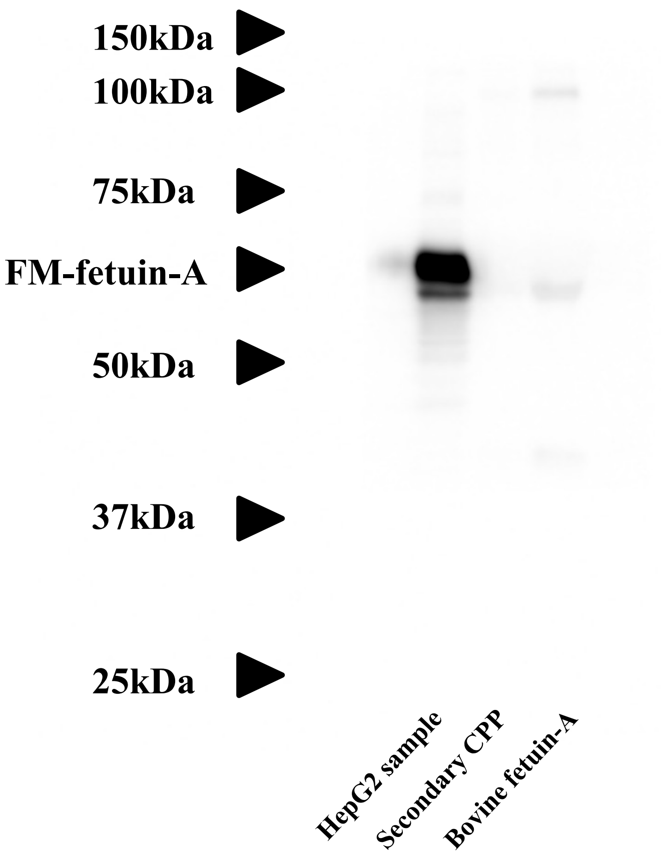

40μg protein loading in each lane

(D') (high-contrast image to make membrane edges visible using same membrane)

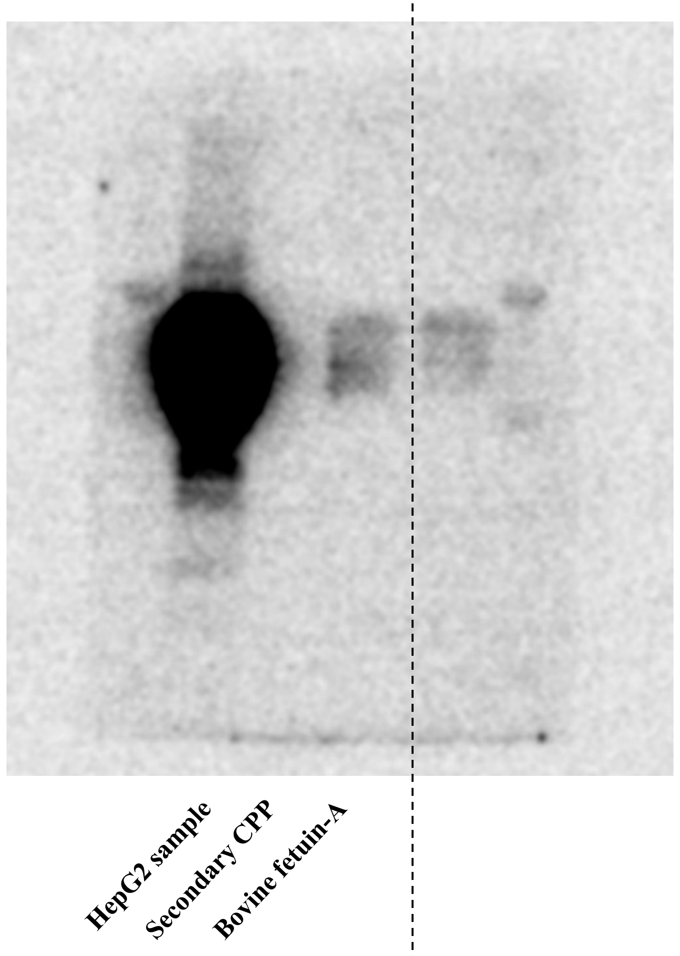

Supplementary Figure X1 (full-size)

(E) (full-length blots)

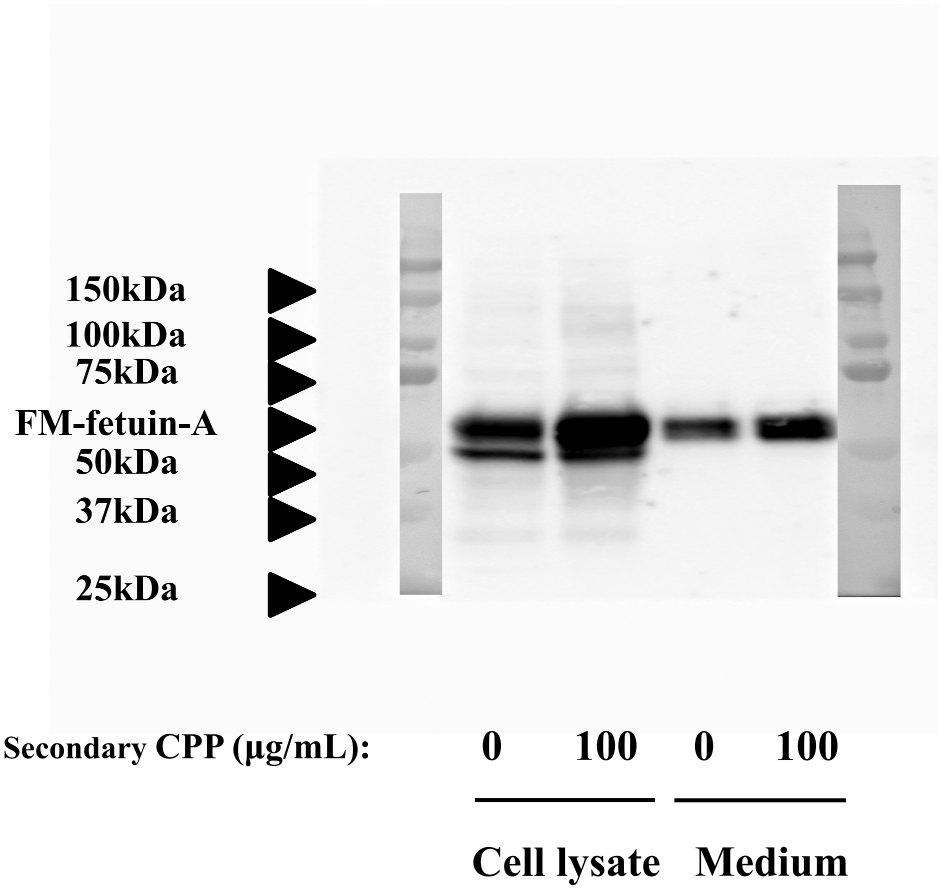

(E') (high-contrast image to make membrane edges visible using same membrane)

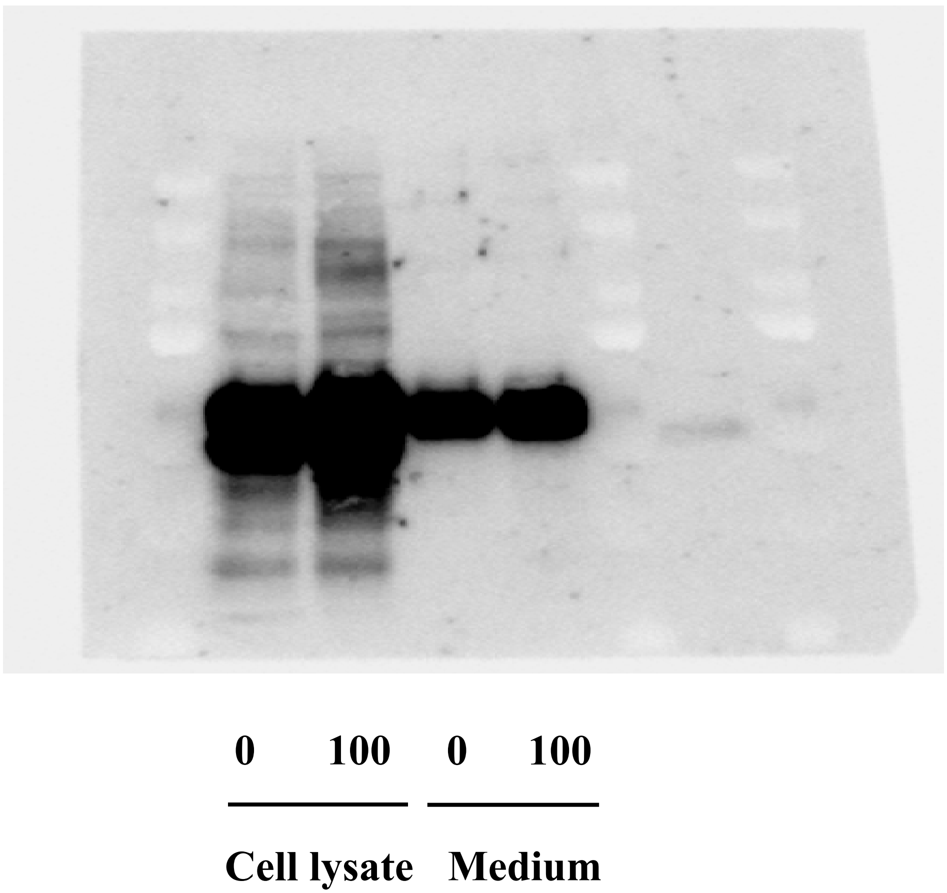

Supplementary Figure X4 (full-size)

(full-length blots)

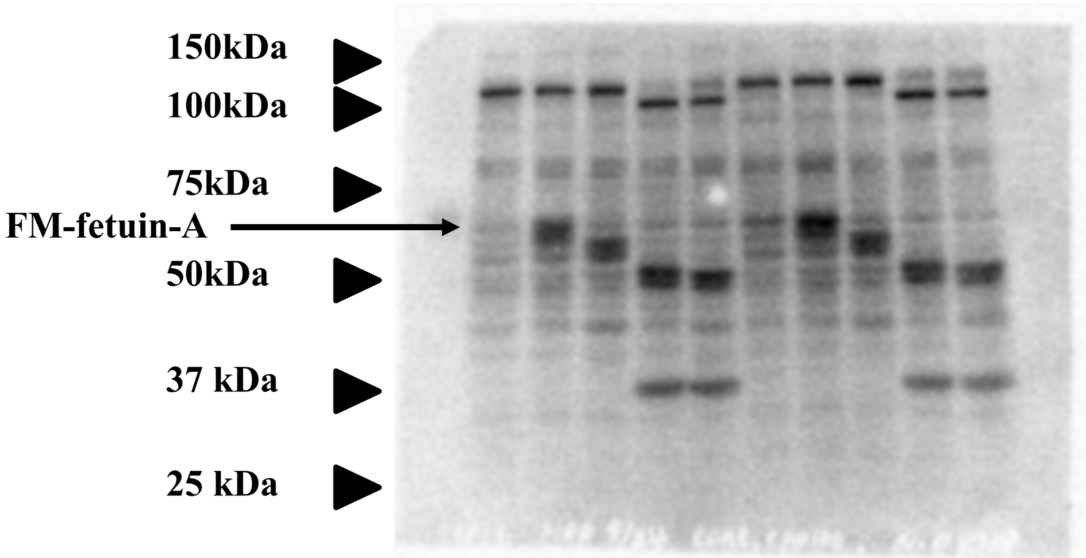

Secondary CPP (µg/mL):	(-)	(+)	(+)	(+)	(+)	(-)	(+)	(+)	(+)	(+)
O-glycosidase:	(-)	(-)	(+)	(-)	(+)	(-)	(-)	(+)	(-)	(+)
N-glycosidase:	(-)	(-)	(-)	(+)	(+)	(-)	(-)	(-)	(+)	(+)

Supplementary Figure X5 (full-size)

(full-length blots)

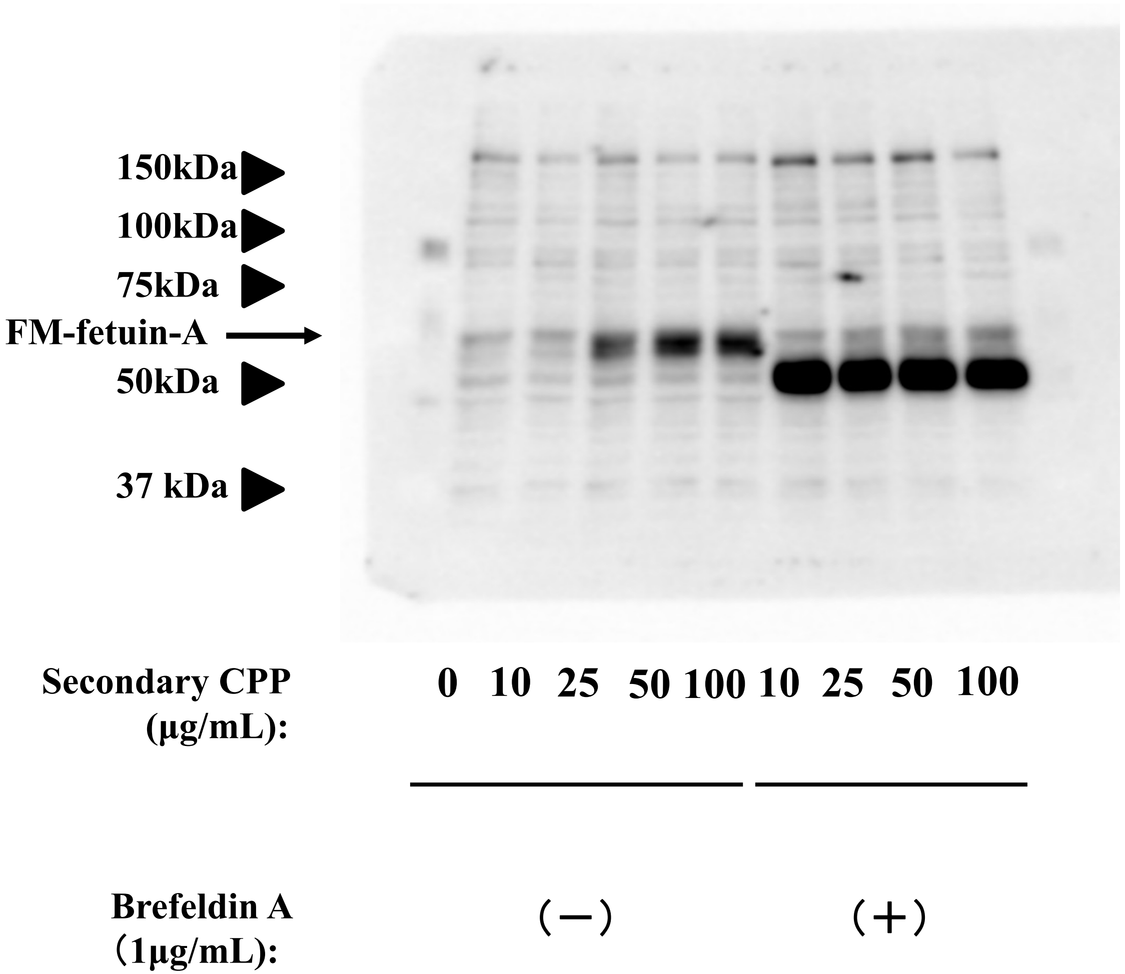

Supplementary Figure X6 (full-size)

(full-length blots)

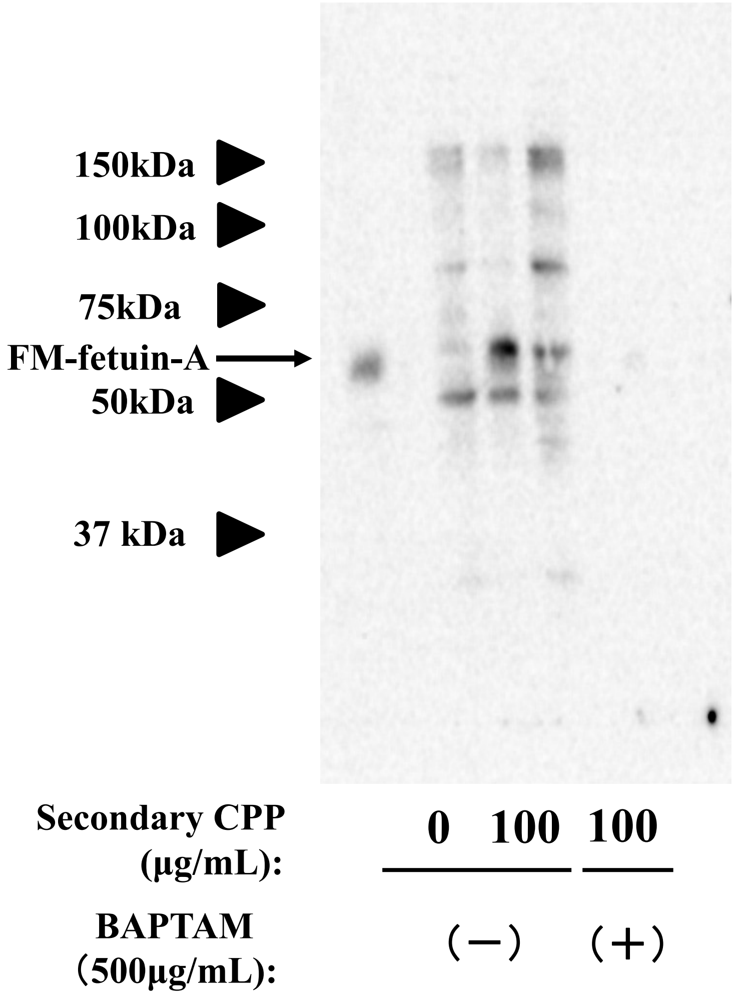

Supplement: Supplementary file 1 — Supplementary Figures. [file 41598_2021_86881_MOESM1_ESM.pdf]
